# The Impact of Collagen Fiber and Slit Orientations on Meshing Ratios in Skin Meshing Models

**DOI:** 10.3390/biomimetics10110771

**Published:** 2025-11-14

**Authors:** Masoumeh Razaghi Pey Ghaleh, Denis O’Mahoney

**Affiliations:** 1 Department of Mechanical and Industrial Engineering, Atlantic Technological University, H91 T8NW Galway, Ireland; 2 Center for Mathematical Modeling and Intelligent Systems for Health and Environment (MISHE), Atlantic Technological University, F91 YW50 Sligo, Ireland

**Keywords:** skin meshing, anisotropic, collagen fibers orientation

## Abstract

Skin meshing facilitates the greater expansion of donor skin through patterned slits and is widely used for treating extensive burn injuries. However, the actual expansion often falls below manufacturers’ claims. Previous computational analyses using the isotropic Yeoh model have shown that Langer’s line orientation and slit direction significantly affect induced stress and meshing ratios, yet the use of nonlinear anisotropic models that represent collagen fiber alignment corresponding to Langer’s lines remains unexplored. This study employs a nonlinear anisotropic Gasser–Ogden–Holzapfel (GOH) model with slit orientations of 0°, 45°, and 90°, consistent with geometries reported in the literature, to quantify induced stress in skin meshing by incorporating collagen fibers within the dermis layer. The GOH parameters were calibrated to human back skin data uniaxially stretched parallel and perpendicular to Langer’s lines using Levenberg–Marquardt optimization in the GIBBON toolbox (MATLAB R2023a) coupled with FEBio v4.0, achieving a standard deviation of 3% relative to experimental data. The GOH model predicted the highest induced stress at 100% strain for the 45° slit parallel to Langer’s lines and the lowest for the 90° slit perpendicular, exceeding 40 MPa due to absence of damage and rupture modeling but accurately representing anisotropic mesh behavior.

## 1. Introduction

In October 2023, the World Health Organization (WHO) reported 180,000 burn-related deaths globally, with the majority occurring in LMICs (low–middle-income countries). Burn injuries are also among the leading causes of disability-adjusted life-years (DALYs) lost in LMICs. DALYs is a metric used to assess the overall disease burden [1,2]. The extent of burns is measured by the percentage of affected total body surface area (TBSA). Larger burns that exceed 20–30% TBSA are considered severe and require medical treatment, such as skin grafting [2,3].

Skin grafting is a surgical procedure in which skin is harvested from a donor site and transplanted to the recipient wound area. It is broadly categorized into traditional sheet graft [4], skin meshing [5], and a more advanced technique known as the meek technique [6]. Skin grafts are generally composed of split-thickness skin grafts (STSG) and full-thickness skin grafts (FTSG), which can be employed in sheet grafts and skin meshing [4,6]. In STSGs, the epidermis and part of the dermis are transplanted into the wound site, whereas in FTSGs, the epidermis and the entire dermis are transplanted [4,6]. STSGs enable resource-efficient coverage of extensive wounds with limited donor skin and are widely used as standard practice in burn management [7,8].

In skin meshing, a skin mesher establishes multiple slits on the skin surface to increase the graft’s meshing ratio and enhance the graft’s capacity for expansion [9]. The meshing ratio is defined as the area of the graft after expansion compared to its original area [10]. However, a discrepancy was observed between the meshing ratios claimed by manufacturers (e.g., 1.5:1, 3:1) and those achieved in practice (e.g., 1.2:1, 1.5:1) [5,9,11,12].

The reason for this divergence in expansion ratios has not been thoroughly investigated in the literature. To explore the reason behind the discrepancies, it is essential to understand the skin’s biology and mechanical properties. Skin is the largest organ in the human body, covering an approximate area of 1.8 m^2^ in adults [13]. It consists of three main layers: the epidermis, dermis, and hypodermis [14]. The epidermis forms a protective barrier against harmful external factors such as heat, infections, and injuries [14,15]. The dermis is composed of collagen and elastin fibers, which govern the strength and stretchability of the skin, respectively [16], while the hypodermis, composed largely of adipose tissue, anchors the skin to the underlying muscles and bones [15,17].

From a biomechanical perspective, Lanir and Fung [18] reported that rabbit skin exhibits nonlinear, anisotropic, and viscoelastic stress–strain behavior under a biaxial tensile loading. Brown et al. [19] further demonstrated that the anisotropic properties of human skin are associated with the collagen network, which consists of undulated fibers that gradually straighten and bear load under increasing tension. Flynn and McCormack [17] also showed that the epidermis and hypodermis exhibit isotropic behavior, whereas the dermis displays anisotropic characteristics due to the orientation and mechanical response of collagen fibers.

Although multi-layered representations of skin have been proposed by Flynn and McCormack [17] and Gupta et al. [12], most computational studies still employ a simplified single-layer model representing the dominant dermal component [10,11,12,20,21,22,23,24,25,26,27,28,29,30,31]. In most skin meshing simulations, isotropic constitutive formulations such as the nonlinear Yeoh model are adopted, which, while suitable for capturing general nonlinear elasticity, neglect the anisotropic characteristics intrinsic to skin mechanics [30,32].

In 2018, Capek et al. [32] employed an isotropic Yeoh model with meshing ratios up to 1:1.5 and emphasized the importance of accounting for Langer’s line while modeling meshing. Capek et al. [32] reported that when identical skin samples are stretched parallel and perpendicular to Langer’s lines, the maximum induced stress is lower in the perpendicular direction, consistent with the directional dependence reported in earlier biaxial tests on rabbit skin by Lanir and Fung [18]. This finding indicates that the stress–stretch response is direction-dependent, reflecting the skin’s anisotropic properties while neglecting the employment of an inherited anisotropic constitutive model [32]. Langer’s lines correspond to the natural tension lines of the skin and align with the predominant orientation of collagen fibers in the dermis, which largely governs skin strength and stiffness [16,33]. Consequently, skin exhibits greater stiffness when stretched along Langer’s lines [18]. Therefore, incorporating a constitutive model that accounts for collagen fiber orientation and dispersion is crucial in computational analyses to accurately capture the mechanical behavior of skin [34].

Among the constitutive models that account for collagen fiber orientation and dispersion, the Gasser–Ogden–Holzapfel (GOH) model was found to be the best fit for the empirical uniaxial tensile data of human back skin reported by Ní Annaidh et al. [34], achieving coefficients of determination of 99.54% parallel and 97.96% perpendicular to Langer’s lines, respectively.

The structural GOH model explicitly links the preferred orientation of collagen fibers to a structural tensor defined by a probability density function (PDF), typically a von Mises distribution. This formulation represents fibers as continuously distributed around a mean orientation, directly reflecting the microstructure of the dermal layer. Allowing slight variations in fiber alignment enables the model to produce a smooth and realistic anisotropic response [35]. Furthermore, as the GOH parameters can be directly correlated with histological measurements, this model demonstrates superior agreement with experimental skin data, particularly at higher strain ratios, relative to traditional isotropic or phenomenological models, such as those proposed by Fung, Ogden, and Mooney–Rivlin [34,36,37,38].

In this paper, the GOH parameters were independently extracted using the inverse Levenberg–Marquardt optimization algorithm available in the GIBBON toolbox [39] in MATLAB R2023a, in conjunction with the open-source software FEBio v4.0, based on the uniaxial tensile (UTS) data reported by Ní Annaidh et al. [34]. The motivation for performing an independent inverse analysis arises from the identical reported values of $c,\;k_{1},$ and $k_{2}$ for two individual samples of 81 and 89 years old, while assigning distinct κ values of 0.1535 and 0.1456, respectively. The UTS was only performed on a single specimen with one specified κ value, which justified the importance of the independent inverse analysis.

The skin meshing geometry was adopted from Gupta et al. [11], featuring slit orientations of 0°, 45°, and 90°, corresponding, respectively, to slits aligned parallel, diagonal, and perpendicular to the stretch direction. The samples were stretched both parallel and perpendicular to Langer’s line directions, and simulations were performed in FEBio v4.0 at strain ratios of 100%, 200%, and 300%. Finally, the induced stresses were evaluated considering both the influence of slit orientation and Langer’s line directions, and the results were compared with those obtained from the isotropic modeling approach proposed by Gupta et al. [11].

This approach facilitates a faster, cost-effective computational design of realistic anisotropic skin meshes while identifying geometries that better resist premature rupture.

## 2. Materials and Methods

**Figure 1:**
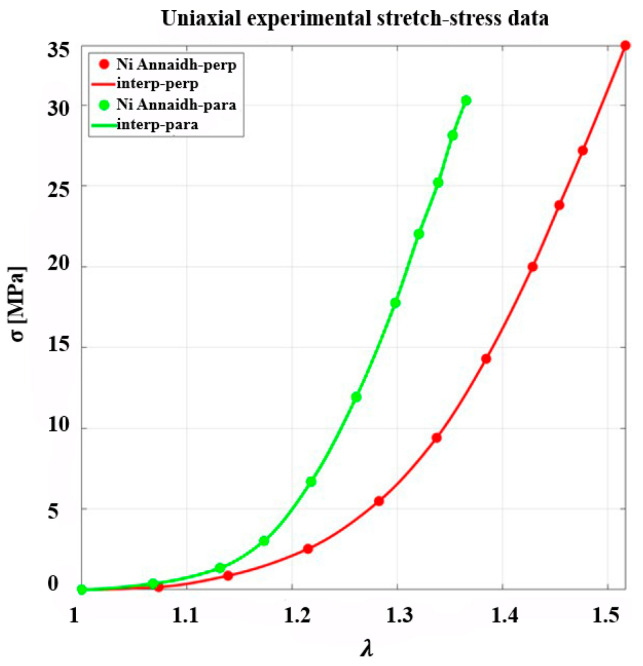
Experimental stress–stretch data of human skin under uniaxial tension applied parallel and perpendicular to Langer’s line, where σ and λ are the principal stress and stretch at 50% strain ratio, respectively (data adapted from Ní Annaidh et al. [34]).

The skin meshing modeling was implemented using a two-step approach. In the first stage, the anisotropic, nonlinear, and uncoupled GOH model was calibrated through inverse analysis of ex vivo uniaxial tensile data for human back skin reported by Ní Annaidh et al. [34], as illustrated in Figure 1. The experimental UTS data, obtained for a specimen stretched up to 50% strain ratios in directions parallel and perpendicular to Langer’s lines, was used to identify the material parameters $c,\;k_{1},\;k_{2},$ and κ simultaneously. The optimization was performed, as detailed in Section 3.2, using the Levenberg–Marquardt algorithm implemented in the GIBBON toolbox [39] (MATLAB R2023a) coupled with FEBio v4.0, while the mean collagen fiber orientation angle was fixed at γ=41°, corresponding to the mean orientation of the two fiber families in human dermis [34].

The uncoupled GOH model was selected for its ability to capture large deformations while maintaining computational stability by enforcing near-incompressibility through a high bulk modulus (K≅1000c), which kept the deformation gradient (J) close to one, thereby achieving near-incompressibility of the skin model [40]. This model is explained in detail in Section Gasser–Ogden–Holzapfel (GOH) Mode.

**Figure 2:**
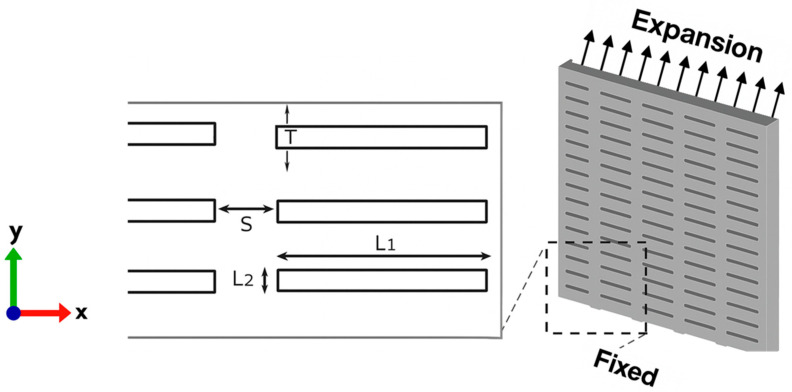
Schematic demonstration of the skin meshing model with boundary conditions.

In the second stage, the extracted GOH parameters were applied in finite element modeling (FEM) to investigate the influence of collagen fibers within the dermis on the skin’s mechanical response and the resulting maximum induced stress, compared with the isotropic Yeoh model used by Gupta et al. [11]. To ensure methodological consistency, the simulations were based on the skin meshing geometry proposed by Gupta et al. [11], considering three slit orientations of 0° (perpendicular), 45° (diagonal), and 90° (parallel) to stretch directions. A 3D model was developed in SolidWorks (Dassault Systèmes, Vélizy-Villacoublay, France) with outer dimensions of 25×25×2 mm, as shown in Figure 2. The slit parameters were defined as $L_{1}=4\;mm,L_{2}=0.4\;mm,T=2mm,$ and S=1mm, representing slit length, height, thickness, and spacing between slits, respectively. The FEM implemented in FEBio v4.0, a standardized biomechanics software, was then used to evaluate the induced stress in samples stretched parallel and perpendicular to Langer’s line.

### Gasser–Ogden–Holzapfel (GOH) Model

The strain energy density function of the anisotropic, nonlinear, uncoupled GOH model, denoted as $\psi_{r}$, represents the energy stored within a material under deformation, enabling the prediction of stress responses in anisotropic, fiber-aligned tissues. The mathematical representation of this model is provided in Equation (1) [35]:

(1) $$\psi_{r}=\frac{c}{2}\left(I_{1}-3\right)-c\ln J+\frac{k_{1}}{2k_{2}}\sum_{\alpha }\left(exp\left(k_{2}{\langle E_{\alpha }\rangle }^{2}\right)-1\right)+\frac{k_{0}}{2}\left(\frac{J^{2}-1}{2}-\ln J\right)$$

where Equation (2) defines the fiber strain

(2) $$E_{\alpha }=\kappa \left(I_{1}-3\right)+\left(1-3\kappa \right)\left(I_{4\alpha }-1\right)$$

In the GOH-unconstrained model, the term $\frac{c}{2}\left(I_{1}-3\right)$ represents the isotropic component of the strain energy associated with the material’s ground matrix. Here, c denotes the shear modulus constant of the ground matrix. $I_{1}$ is the first invariant of the deviatoric part of the right Cauchy–Green deformation tensor C, defined as $I_{1}=tr\;C$. The invariant $I_{4\alpha }$ corresponds to the direction of the fiber within the material and captures the anisotropic contribution toward the strain energy. The fiber direction vector in the reference configuration is denoted as $a_{\alpha r}$. $I_{4\alpha }$ and is also mathematically expressed as ${I_{4\alpha }=a}_{\alpha r}\cdot C\cdot a_{\alpha r}$, where the dot products describe the interaction between the fiber direction and the deformation tensor.

**Figure 3:**
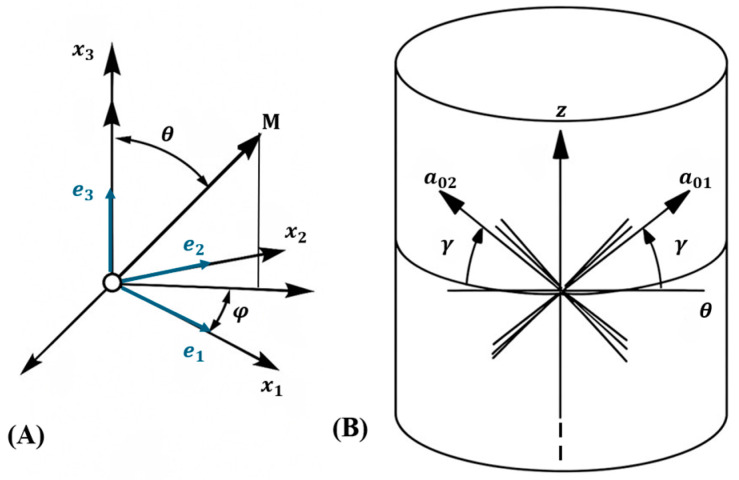
(**A**) Characterization of an arbitrary unit direction of a vector M by means of Eulerian angles $\Phi \in \left[0,2\pi \right]$ in a three-dimensional Cartesian coordinate system $\left\{e_{1},\;e_{2},\;e_{3}\right\}$. (**B**) The orientation of the families of fibers is shown in a two-dimensional cylindrical coordinate system. γ indicates the angle of fibers with $e_{1}$ axis.

In the model, two fiber families are oriented along the vectors $a_{\alpha r}\;(\alpha =1,\;2)$, which lie within the $\left\{e_{1},e_{2}\right\}$ plane of the local material axes $\left\{e_{1},\;e_{2},\;e_{3}\right\}$. These fibers are aligned at angles ±γ relative to $e_{1}$, as depicted in Figure 3. Each fiber family exhibits a dispersion characterized by κ, where $0\leq \kappa \leq \frac{1}{3}$. When κ=0, there is no fiber dispersion, indicating that all fibers in the family are perfectly aligned at angles ±γ. In contrast, $\kappa =\frac{1}{3}$ corresponds to isotropic fiber dispersion, where fibers are uniformly distributed without preferential alignment. Intermediate values of κ describe a periodic von Mises fiber distribution with a periodicity of π, reflecting varying degrees of alignment and dispersion within the fiber family.

Here, $a_{1}$ and $a_{2}$ are unit vectors representing the preferred orientations of two families of collagen fibers within the tissue, and are described as $a_{1}=cos\gamma i+sin\gamma j$ and $a_{2}=cos\gamma i-sin\gamma j$.

In Equation (1), the term −clnJ provides an additional isotropic contribution to the strain energy, which is associated with volume changes and is added to control compressibility. Although the unconstrained GOH model does not strictly enforce incompressibility, this term adjusts the energy related to volume change, enabling the model to exhibit near-incompressibility when *c* is sufficiently large. Here, *J*, the determinant of the deformation gradient, represents the volume change, with J≈1 indicating that the material behaves in a nearly incompressible fashion.

The term $\frac{k_{1}}{2k_{2}}\sum_{\alpha }\left(exp\left(k_{2}{\langle E_{\alpha }\rangle }^{2}\right)-1\right)$ represents the anisotropic contribution from collagen fibers. In this expression, $k_{1}$ and $k_{2}$ are material parameters where $k_{1}$ represents the fiber modulus and $k_{2}$ represents the fiber exponential coefficient, describing fiber stiffness in the small strain regime and large strain stiffening behavior, respectively [34]. The Macaulay brackets $\langle E_{\alpha }\rangle$ are used to account for directional effects, such that $\langle E_{\alpha }\rangle =0$ when $E_{\alpha }<0$, and $\langle E_{\alpha }\rangle =E_{\alpha }$ when $E_{\alpha }$ is positive, ensuring that only tensile strains contribute to the anisotropic energy.

Finally, the term $\frac{k_{0}}{2}\left(\frac{J^{2}-1}{2}-\ln J\right)$ represents a volumetric penalty that governs volume changes, facilitating near-incompressibility in the model. The parameter $k_{0}$ corresponds to the bulk modulus, which determines the material’s volumetric response in the unconstrained model. According to the FEBio documentation and literature [40], for achieving near-incompressibility of biological tissue, $k_{0}$ is recommended to be $k_{0}≅1000c$, where *c* is the shear modulus.

## 3. Results and Discussion

This section presents the inverse analysis, finite element modeling, and results in three parts. Initially, Section 3.1 describes the steps in inverse analysis used to calibrate the uncoupled GOH model parameters from experimental tensile data. Next, Section 3.2 outlines the skin meshing geometry’s finite element simulations and convergence analysis. Finally, Section 3.3 discusses the results, highlighting the effects of collagen fiber orientation and slit geometry on the anisotropic mechanical response of skin.

### 3.1. Inverse Analysis of Uncoupled GOH Model

To verify the constitutive response of human skin, the uniaxial stress–stretch behavior reported by Ní Annaidh et al. [34] was re-examined in FEBio v4.0. The originally published material parameters failed to reproduce the experimental results, necessitating an independent inverse analysis for parameter calibration. In the study by Ní Annaidh et al. [34], the dispersion factor κ, which quantifies the distribution of collagen fiber orientations through a probability density function, was computed using the numerical integration approach described by Gasser et al. [35]. The κ values were obtained from two different donor samples (81- and 89-year-old individuals), although the uniaxial tensile test (UTS) was conducted on only one specimen, as illustrated in Figure 1.

This inconsistency suggested an overparameterized calibration process and potential misrepresentation of collagen fiber dispersion. To ensure physical and mathematical consistency, the mean collagen fiber orientation (γ = 41°), corresponding to the mean orientation of two fiber families in human dermis, was fixed throughout our optimization.

**Figure 4:**
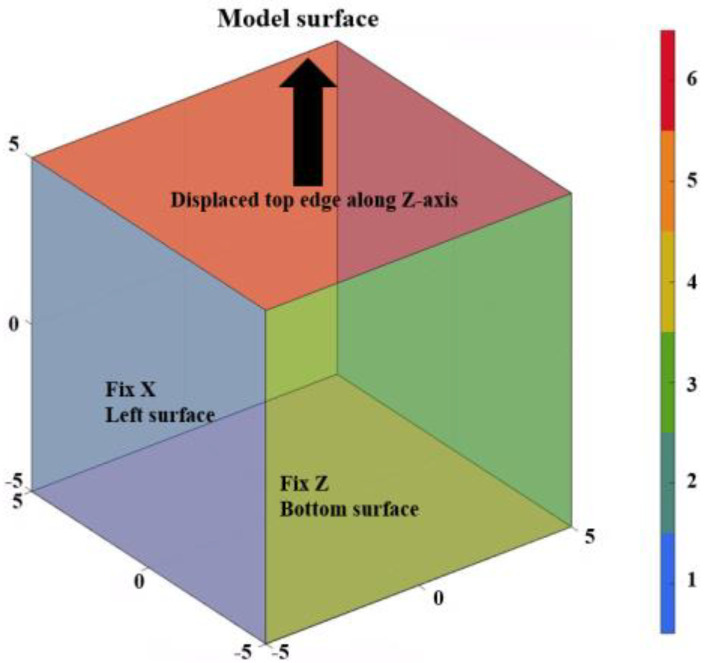
Boundary conditions for the single element used in the curve fitting of the uncoupled GOH model.

The material constants *c*, *k*_1_, *k*_2_, and κ were optimized simultaneously using the Levenberg–Marquardt algorithm implemented in the GIBBON toolbox [39] (in MATLAB R2023a) coupled with FEBio v4.0, for the sample stretched both parallel and perpendicular to Langer’s lines. The Levenberg–Marquardt method combines the robustness of gradient descent and the efficiency of the Gauss–Newton approach, offering superior convergence for nonlinear least-squares optimization, particularly in hyperelastic constitutive modeling [41]. A single cubic element (10×10×10 mm) was analyzed under uniaxial displacement boundary conditions: the left, back, and bottom faces were fixed in the X, Y, and Z directions, respectively, while a 5 mm displacement was applied on the top surface, corresponding to a 50% strain ratio, as shown in Figure 4.

**Figure 5:**
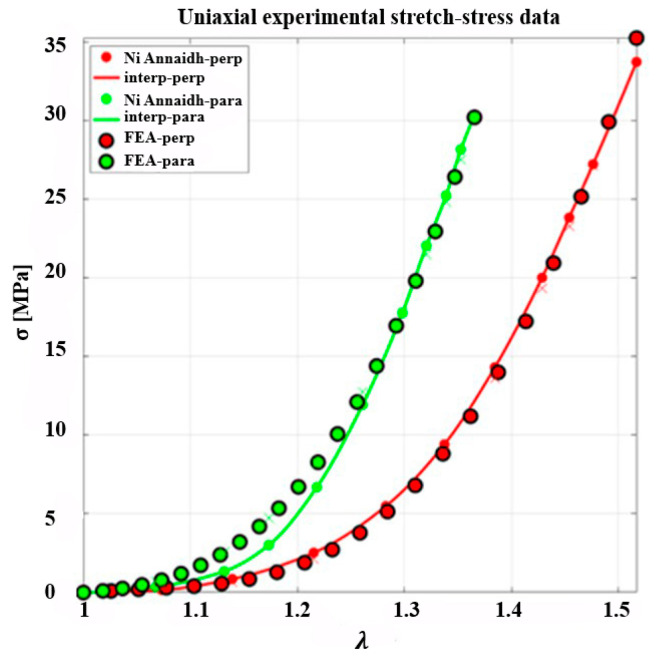
Comparison between the experimental stress–stretch data reported by Ní Annaidh et al. [36] and the fitted curves obtained using the Levenberg–Marquardt optimization algorithm with uncoupled GOH model parameters.

Using this approach, the parameters were optimized with an initial guess based on Ní Annaidh et al. [34], while ensuring near-incompressibility with K≅1000c, consistent with incompressibility criteria reported in the literature [40]. The optimization achieved a sum of squared errors (SSE) of 12.39 for both parallel and perpendicular directions, and a standard deviation (SD) of 3%, indicating excellent agreement with the experimental stress–stretch data, as shown in Figure 5. The optimized parameters are presented in Table 1.

In the GOH model, $k_{1}$ governs the fiber stiffness and resistance to stretching along the preferred directions, whereas $k_{2}$ defines the exponential stiffening of collagen fibers at large strains. The obtained small $k_{2}$ value suggests a gradual stiffening response that is consistent with the nonlinear behavior of dermal collagen networks. These calibrated parameters were subsequently applied in the FEM simulations described in Section 3.2.

### 3.2. Finite Element Modeling and Convergence Analysis

Finite element simulations were performed in FEBio v4.0 to evaluate the induced stresses under uniaxial stretching conditions. The model geometry was adopted from Gupta et al. [11], consisting of a 25×25×2 mm skin specimen incorporating slit orientations of 0° (horizontal), 45° (diagonal), and 90° (vertical) relative to the stretch direction. The bottom surface was constrained in all degrees of freedom (*X*, *Y*, *Z*), while the top surface was displaced along the *Y*-axis (Figure 2) by 25 mm, 50 mm, and 75 mm; corresponding to nominal stretch ratios of 100%, 200%, and 300%, respectively. This setup simulated a clinical skin meshing scenario in which the graft is stapled at one end and stretched longitudinally to cover a wound area [42].

**Figure 6:**
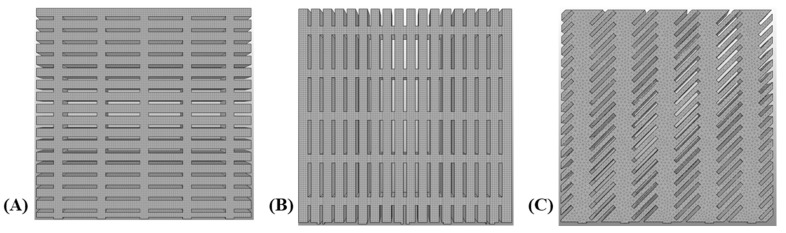
Schematic of skin meshing: (**A**) horizontal slits with hexahedral elements, (**B**) vertical slits with hexahedral elements, and (**C**) 45° slits with tetrahedral elements.

**Figure 7:**
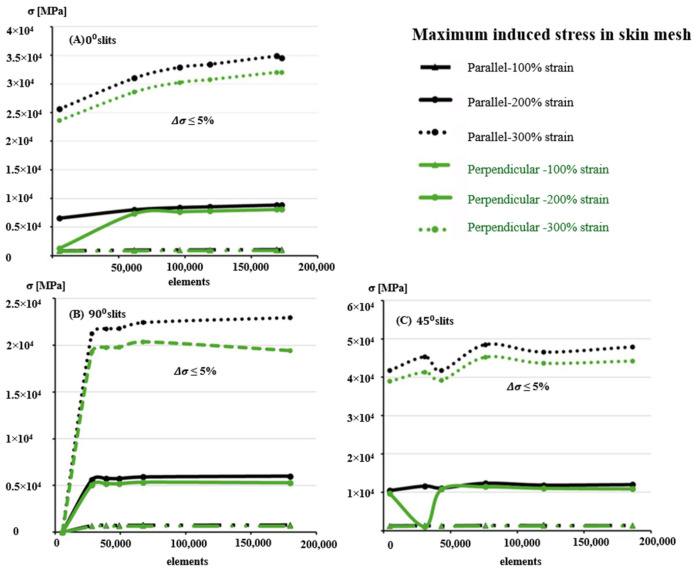
Maximum induced stress as a function of mesh density for skin models with stretch ratios of 100%, 200%, and 300%. Results are shown for three slit orientations: (**A**) horizontal (0°), (**B**) vertical (90°), and (**C**) 45° patterns. Convergence tests indicate a numerical error below ±5%, confirming the stability of the finite element results across mesh refinements.

**Figure 8:**
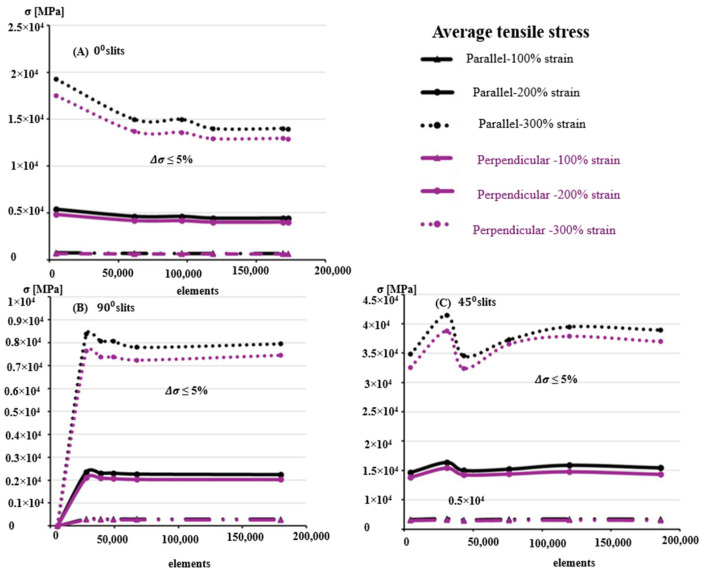
Average tensile stress as a function of mesh density for skin models with stretch ratios of 100%, 200%, and 300%. Results are presented for three slit orientations: (**A**) horizontal (0°), (**B**) vertical (90°), and (**C**) 45° patterns. Convergence analysis shows that the numerical error remains below ±5%, demonstrating the reliability of the finite element results across different mesh refinements.

To enhance computational stability and accuracy, hexahedral 8-node elements were employed for the 0° and 90° slit orientations, while tetrahedral 10-node elements were used for the 45° case to better capture stress gradients around sharp slit corners, as shown in Figure 6. Mesh convergence was evaluated using coarse, medium, and fine meshes ranging from approximately 5000 to 186,000 elements. Convergence was achieved when the relative change in maximum induced stress fell below ±5%, corresponding to 169,213 elements for horizontal, 67,480 for vertical, and 119,654 for 45° slit geometries. Figure 7 and Figure 8 illustrate the representative meshing strategies and the corresponding convergence trends.

The computed stress–strain responses were extracted for the overall geometry and the top surface to support future validation through uniaxial tensile testing (UTS) of samples stretched parallel and perpendicular to Langer’s lines. The consistency of stress results across increasing mesh densities confirmed the robustness of the GOH-based finite element implementation and its suitability for analyzing slit-induced anisotropy in skin meshing.

### 3.3. Influence of Anisotropy and Slit Orientation

The calibrated anisotropic GOH model successfully captured the directional dependence of skin stiffness along Langer’s lines. As expected, for the same nominal strain, samples stretched parallel to Langer’s lines exhibited higher stress values than those stretched perpendicular, confirming the anisotropic mechanical response arising from collagen fiber alignment [18,19,34].

**Figure 9:**
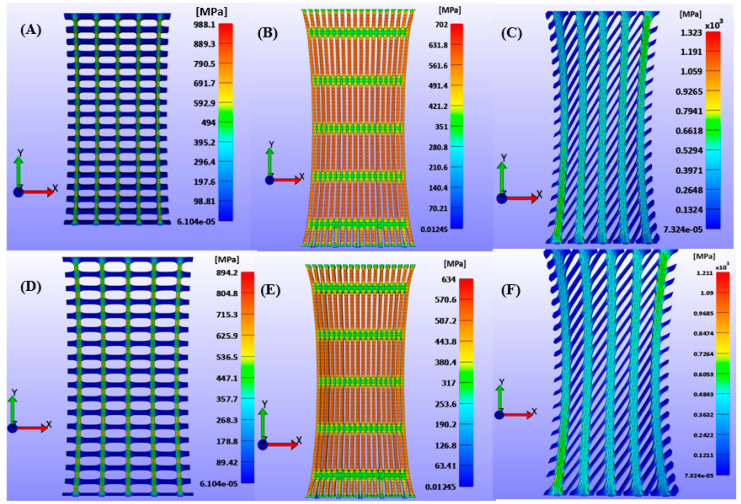
Maximum principal stress of skin meshing model with varying slit angles, and when the skin is stretched with stretch ratios of 100%, parallel and perpendicular to the direction of Langer’s line: (**A**) 0°-parallel fibers, (**B**) 90°-parallel fibers, (**C**) 45°-parallel fibers, (**D**) 0°-perpendicular fibers, (**E**) 90°-perpendicular fibers, and (**F**) 45°-perpendicular fibers.

Across all three slit orientations (0°, 45°, and 90°) and stretch ratios (100%, 200%, and 300%), the 45° diagonal slits produced the highest induced stress, both in terms of maximum total induced stress and average surface stress, whereas the 90° vertical slits generated the lowest (Figure 9). This trend reflects the coupling between slit geometry and fiber orientation: diagonal slits promote greater local fiber stretching and stress concentration, whereas vertical slits align with the dominant load path, reducing stress accumulation.

**Figure 10:**
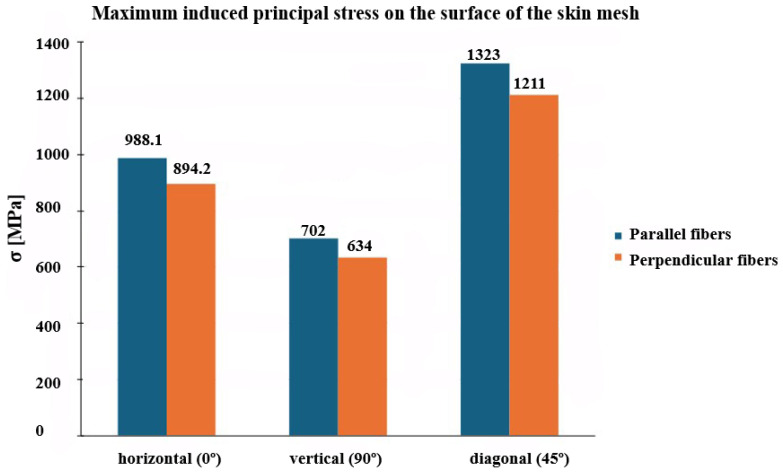
Comparison of principal stress induced by an anisotropic nonlinear model for both parallel and perpendicular fiber directions to stretch for three slit patterns of vertical, horizontal, and 45° slit patterns with stretch ratios of 100%.

Notably, the maximum induced principal stress exceeded the ultimate tensile strength (UTS) of human skin (≈40 MPa [34]), consistent with the absence of damage or rupture modeling in the GOH formulation, as shown in Figure 10.

Quantitatively, at a 100% stretch ratio, reducing the slit angle from 90° to 45° increased the maximum induced stress by approximately 88%, while further reduction to 0° decreased it by about 34%. In contrast, Gupta et al. [11], using the isotropic Yeoh model, reported only minor stress variations (≈0.1%) across slit orientations, with an opposite trend where vertical slits exhibited slightly higher stresses than horizontal ones. Further reducing the slit angle from 45° to 0° in the Yeoh model increased stress by about 60%.

These observations highlight the limitations of isotropic formulations in capturing the directional dependence of skin behavior and underscore the value of anisotropic models. The GOH model provides a more realistic prediction of stress distribution and deformation patterns by accounting for the fiber-reinforced architecture of the dermis. This finding is consistent with previous studies demonstrating that collagen fiber orientation governs the anisotropic stiffness and load-bearing capacity of skin [18,19,34].

Although the current implementation does not include a damage formulation, it effectively demonstrates that slit geometry and Langer’s line orientation jointly dictate the local stress state in skin meshing. These insights are critical for optimizing graft designs to minimize premature rupture and improve expansion efficiency.

## 4. Conclusions

This study compared how the integration of the anisotropic nonlinear GOH model, incorporating embedded collagen fibers, and slit variation under a uniform strain ratio of 100% influences the induced stress, relative to the isotropic Yeoh model of skin meshing. Furthermore, the effect of Langer’s line orientation was examined for parallel (0° horizontal), vertical (90°), and diagonal (45°) slit configurations. The results showed that skin meshing designs with 90° slit orientations exhibited the lowest induced stress values, whereas the 45° diagonal orientations produced the highest stress at ultimate tensile strain, indicating a greater likelihood of early damage under loading conditions. These findings, which contrast with previous isotropic analyses, emphasize the importance of accounting for both Langer’s line orientation and geometry in modeling skin meshing. The main limitation of this study lies in the absence of a GOH-based damage formulation and experimental UTS validation, both of which are crucial for strengthening the computational framework and improving the reliability of predictions. The development and implementation of a GOH-based damage model will be the focus of future work to capture rupture behavior and enhance the predictive accuracy of skin meshing design.

## Figures and Tables

**Table 1:** Obtained values through inverse analysis of the GOH parameters.

Constitutive Model	c [MPa]	$k_{1}$	$k_{2}$	γ	κ	K
Parallel and Perpendicular	1.30259	124.351	0.00013	41°	0.2985	1302.59

## Data Availability

The original data and code used in this study are based on resources available in GibbonCode (www.gibboncode.org). The specific dataset DEMO_febio_0089_iFEA_goh_skin_01 can be requested from the corresponding author and will be made openly accessible on the GibbonCode documentation page upon publication of the related companion study.

## References

[1] Burns. https://www.who.int/news-room/fact-sheets/detail/burns.

[2] Peck M.D. (2011). Epidemiology of Burns throughout the World. Part I: Distribution and Risk Factors. Burns.

[3] Radzikowska-Büchner E., Łopuszyńska I., Flieger W., Tobiasz M., Maciejewski R., Flieger J. (2023). An Overview of Recent Developments in the Management of Burn Injuries. Int. J. Mol. Sci..

[4] Kohlhauser M., Luze H., Nischwitz S.P., Kamolz L.P. (2021). Historical Evolution of Skin Grafting—A Journey through Time. Medicina (B Aires).

[5] Vandeput J., Nelissen M., Tanner J.C., Boswick J. (1995). A Review of Skin Meshers. Burns.

[6] Singh M., Nuutila K., Collins K.C., Huang A. (2017). Evolution of Skin Grafting for Treatment of Burns: Reverdin Pinch Grafting to Tanner Mesh Grafting and Beyond. Burns.

[7] Noureldin M.A., Said T.A., Makeen K., Kadry H.M. (2022). Comparative Study between Skin Micrografting (Meek Technique) and Meshed Skin Grafts in Paediatric Burns. Burns.

[8] Wainwright D.J. (2009). Burn Reconstruction: The Problems, the Techniques, and the Applications. Clin. Plast. Surg..

[9] Lyons J.L., Kagan R.J. (2014). The True Meshing Ratio of Skin Graft Meshers. J. Burn. Care Res..

[10] Gupta V., Singh G., Chanda A. (2023). Development of Novel Hierarchical Designs for Skin Graft Simulants with High Expansion Potential. Biomed. Phys. Eng. Express.

[11] Gupta V., Singh G., Chanda A. (2022). Development and Testing of Skin Grafts Models with Varying Slit Orientations. Mater. Today: Proc..

[12] Gupta S., Gupta V., Chanda A. (2022). Biomechanical Modeling of Novel High Expansion Auxetic Skin Grafts. Int. J. Numer. Methods Biomed. Eng..

[13] Singh G., Chanda A. (2021). Mechanical Properties of Whole-Body Soft Human Tissues: A Review. Biomed. Mater..

[14] Hendriks F.M., Brokken D., Oomens C.W.J., Bader D.L., Baaijens F.P.T. (2006). The Relative Contributions of Different Skin Layers to the Mechanical Behavior of Human Skin in Vivo Using Suction Experiments. Med. Eng. Phys..

[15] Tortora G.J., Derrickson B.H. (2017). Principles of Anatomy and Physiology.

[16] Wilkes G.L., Brown I.A., Wildnauer R.H. (1973). The Biomechanical Properties of Skin. CRC Crit. Rev. Bioeng..

[17] Flynn C.O., McCormack B.A.O. (2009). A Three-Layer Model of Skin and Its Application in Simulating Wrinkling. Comput. Methods Biomech. Biomed. Eng..

[18] Lanir Y., Fung Y.C. (1974). Two-Dimensional Mechanical Properties of Rabbit Skin—II. Experimental Results. J. Biomech..

[19] Brown I.A. (1973). A Scanning Electron Microscope Study of the Effects of Uniaxial Tension on Human Skin. Br. J. Dermatol..

[20] Gupta V., Chanda A. (2022). Biomechanics of Skin Grafts: Effect of Pattern Size, Spacing and Orientation. Eng. Res. Express.

[21] Chanda A., Gupta V., Gupta S. (2024). Research on the Application of Auxetics in Skin Grafts.

[22] Gupta V., Gupta S., Chanda A. (2022). Expansion Potential of Skin Grafts with Novel Rotating-Triangle-Shaped Auxetic Incisions. Emerg. Mater. Res..

[23] Gupta V., Chanda A. (2022). Expansion Potential of Skin Grafts with Alternating Slit Based Auxetic Incisions. Forces Mech..

[24] Gupta V., Chanda A. (2023). Auxetic Incisions with Alternating Slit Shapes: A Promising Technique for Enhancing Synthetic Skin Grafts Expansion. Mater. Res. Express.

[25] Gupta V., Singh G., Chanda A. (2023). Modeling of Metamaterial Based Incision Patterns for Generating High Expansions in Skin Grafts. Clin. Biomech..

[26] Gupta V., Chanda A. (2022). Expansion Potential of Skin Grafts with Novel I-Shaped Auxetic Incisions. Biomed. Phys. Eng. Express.

[27] Singh G., Gupta V., Chanda A. (2022). Mechanical Characterization of Rotating Triangle Shaped Auxetic Skin Graft Simulants. Facta Univ. Ser. Mech. Eng..

[28] Gupta V., Singh G., Chanda A. (2023). High Expansion Auxetic Skin Graft Simulants for Severe Burn Injury Mitigation. Eur. Burn. J..

[29] Gupta V., Singh G., Chanda A. (2023). Development of Hierarchical Auxetic Skin Graft Simulants with High Expansion Potential. Biomed. Eng. Adv..

[30] Gupta V., Singla R., Chanda A. (2023). Development and Characterization of Novel Anisotropic Skin Graft Simulants. Dermato.

[31] Gupta V., Chanda A. (2023). Finite Element Analysis of Hierarchical Metamaterial-Based Patterns for Generating High Expansion in Skin Grafting. Math. Comput. Appl..

[32] Capek L., Flynn C., Molitor M., Chong S., Henys P. (2018). Graft Orientation Influences Meshing Ratio. Burns.

[33] Humbert P., Fanian F., Maibach H.I., Agache P. (2017). Agache’s Measuring the Skin Non-Invasive Investigations, Physiology, Normal Constants.

[34] Ní Annaidh A., Bruyère K., Destrade M., Gilchrist M.D., Maurini C., Otténio M., Saccomandi G. (2012). Automated Estimation of Collagen Fibre Dispersion in the Dermis and Its Contribution to the Anisotropic Behaviour of Skin. Ann. Biomed. Eng..

[35] Gasser T.C., Ogden R.W., Holzapfel G.A. (2006). Hyperelastic Modelling of Arterial Layers with Distributed Collagen Fibre Orientations. J. R. Soc. Interface.

[36] Bellini C., Glass P., Sitti M., Di Martino E.S. (2011). Biaxial Mechanical Modeling of the Small Intestine. J. Mech. Behav. Biomed. Mater..

[37] Shergold O.A., Fleck N.A., Radford D. (2006). The Uniaxial Stress versus Strain Response of Pig Skin and Silicone Rubber at Low and High Strain Rates. Int. J. Impact Eng..

[38] Aldieri A., Terzini M., Bignardi C., Zanetti E.M., Audenino A.L. (2018). Implementation and Validation of Constitutive Relations for Human Dermis Mechanical Response. Med. Biol. Eng. Comput..

[39] Moerman K.M. (2018). GIBBON: The Geometry and Image-Based Bioengineering Add-On. J. Open Source Softw..

[40] Nolan D.R., Gower A.L., Destrade M., Ogden R.W., McGarry J.P. (2014). A Robust Anisotropic Hyperelastic Formulation for the Modelling of Soft Tissue. J. Mech. Behav. Biomed. Mater..

[41] Gavin H. (2019). The Levenberg-Marquardt Algorithm for Nonlinear Least Squares Curve-Fitting Problems. Dep. Civ. Environ. Eng. Duke Univ. August.

[42] Taylor B.C., Triplet J.J., Wells M. (2021). Split-Thickness Skin Grafting: A Primer for Orthopaedic Surgeons. J. Am. Acad. Orthop. Surg..

